# A Trustworthy Combination of Coca, Viburnum and Celery, Etc.

**Published:** 1883-07-20

**Authors:** William B. Hazard

**Affiliations:** Professor of General Pathology and of Diseases of the Mind and Nervous System, St. Louis College of Physicians and Surgeons, etc.


### A TRUSTWORTHY COMBINATION OF COCA, VIBURNUM AND CELERY, WITH NOTES OF CASES.

By WILLIAM B. HAZARD, M. D.,

P.ofessor of General Pathology and of D seases of the Mind and Nervous System, St. Louis College of Physicians and Surgeons, etc.

Erythroxylon Coca is a shrub growing in several of the States of South America. The leaves are the parts of the plant used as a nervous stimulant or tonic. A large amount is collected annually by the natives and preserved for use by being dried in the sun. They are oval in shape, about two inches in length, and have a bitter and pungent taste when green; when dried, they taste like Chinese tea. The natives' method of use is by chewing the leaves mixed with a little ashes or lime.

The leaves contain coca-tannic acid, a variety of tannin which gives a green reaction with salts of iron; cocaina, a fixed, crystalizable alkaloid which benumbs the tongue and has a bitter taste; and hygrina, a volatile alkaloid, pale yellow in color, oily in consistence and possessing an alkaline reaction and a burning taste.

The physiological actions of coca upon animals are as follows: In small doses it dilates the pupils, causes hyperæsthesia and loss of co-ordination of movements. In medium doses, it lessens, and then abolishes sensibility. In large doses, it produces tetanic convulsions.

In man, all authorities agree that it prevents fatigue and enables the user to perform more labor with less exhaustion than would otherwise ensue. The natives of South America, at the time of the Spanish conquest, claimed for it a divine origin. This caused the conquerors to interdict its use; but these religious scruples gave way before the fact that the enslaved populace could perform much more labor with than without it—a strong testimonial to the benefits it confers upon its consumers. The results of experiments made in this country are that coca retards, in some way as yet unexplained, tissue metamorphosis, and consequently diminishes waste, while, at the same time, it stimulates the nervous system without subsequent ill effects.

The medicinal virtues of coca are faithfully outlined in its physiological action. It is indicated in all functional affections in which it is a desideratum to prevent waste of muscular or nervous tissue. In all such conditions this agent becomes a valuable aid to conservative medicine. More effective than tea or coffee, and devoid of the per..icious properties of alcohol or opium, it gives the patient a respite from suffering and gives the physician what he most needs: time in which to obtain the reparation of worn-out physiological units of the organism through the agency of improved nutrition.

In the treatment of the alcohol and opium habits, coca has proven itself simply invaluable. Nervous exhaustion, whether superinduced by too prolonged brain-work, business worry, or excesses in venery, finds in coca a valuable help toward a restoration to the normal condition. In convalescence from acute diseases, this again becomes a valuable aid by hindering excessive waste of the body and giving time for the digestive organs to supply new material for the restoration of the exhausted organism.

There is a large class of cases which require something more than a mere nervous stimulant, although the principal symptoms are those of nervous exhaustion. Reference is here made to those instances of disease, which in the female are accompanied with pelvic pain, and, in the male, with urethral and prostatic hyperæsthesia, and in both sexes with depression of spirits amounting to hypochondria reaching almost to melancholia. These cases require a sedative effect to be exerted especially over the reflex apparatus of the genito-spinal and abdominal sympathetic systems.

The varieties of Viburnum (V. opulus and V. prunifolium) have the power of reducing hyperæsthesia and reflex excitability—in these directions. Ovarian neuralgia, tending to abortion and dysmenorrhœa in the woman, and prostatorrhœa, psychical impotency and numberless symptoms of exaggerated irritability of the sensory nerves in the man, are happily relieved by preparations of viburnum.

Celery (Apium graveolens) has long been recognized as possessing strong powers as a diuretic, at the same time controlling irritability of the bladder, and exerting a most beneficial influence over headaches of a nervous origin. As a soothing agent in sleeplessness originating in over-work, and as a stomachic tonic, when taken in moderate quantity after meals, aiding in digestion and assimilation, popular favor has long since marked it out as "a friend in need" to the nervous dyspeptic as well as to those whose appetite tempts to over-indulgence at the table. Celery, when taken in substance, or if the stalks are not properly blanched, often acts disadvantageously, especially if it is hastily swallowed without proper mastication. This may be avoided by the use of a fluid extract. The bane of American life is dyspepsia, which has gained recognition as *the* American disease. Good cooking and sufficient mastication of food would do much to overcome this national enemy. But we have to take things as we find them, and the average American has to have a stomachic stimulant or tonic furnished him to overcome the effects of a bad *cuisine* and worse habits as regards the pursuit of business as well as ingestion of food.

In Richardson's "CELERINA" we find a combination which very fairly meets the requisites hereinbefore pointed out. As a tonic and soothing agent, acting primarily upon the digestive and genito-urinary systems, and as a modifier of the nutrition of the nerves supplying the pelvis, it is, theoretically, well calculated to meet a large class of indications. Practically, it has proven itself worthy of a more extensive trial than it has yet had from the medical profession.

My own experience with "Celerina" has, thus far, been rather limited. In two cases of dysmenorrhœa, accompanied with ovarian hyperæsthesia, due to excessive child-bearing and over-work at the wash-tub, and consequent loss of appetite and emaciation, "Celerina" afforded marked relief. These were charity cases and, consequently, not very favorable subjects for treatment. Under better circumstances, I am satisfied that permanent relief would have resulted.

A case of the most marked nervous exhaustion, consequent on prolonged masturbation, resulting in temporary impotency, was referred to me by a physician in Central Texas, in the summer of 1882. The patient was a very intelligent gentleman of thirty-two years of age ; single ; hypochondriacal, and low-spirited, almost to the verge of suicide. Treatment by strychnia, phosphorus, laxatives and local applications to the neighborhood of the prostate gave him no relief. Cold baths and a general tonic regimen were equally without result. He was finally given two pounds of "Celerina," with directions to take two teaspoonfuls after each meal, which were faithfully carried out, and a permanent cure was effected.

The first result to be achieved in the treatment of such a case, is to secure complete abstinence from the vile practice which was the primary cause of the trouble. This is often beyond the enfeebled powers of the patient, unless he has proper medical aid. I think the effects of viburnum, in this regard, by soothing the urethral mucous membrane and counteracting the "irritable weakness" of the genito-spinal center, may be safely relied upon in cases where there is any amount of will-power available.

In a second case referred to me in October, 1882, by a physician of Southern Kansas, there was less marked hypochondria, due to indigestion, constipation, and lack of out-door exercise. Here a proper laxative, horseback exercise, and regulated diet produced some improvement,but the mental depression was not relieved until the second eight ounces of "Celerina" had been nearly exhausted.

In February of this year, an old gentleman from Southern Arkansas, the subject of attacks of melancholia recurring every two or three years, presented himself to me for treatment. His then present attack was marked by no definite delusion, but by such a profound depression of spirits that he could scarcely refrain from committing suicide whenever the surrounding circumstances would give him the opportunity to take his own life. Although a man of wealth, he feared impending poverty, at the same time recognizing the folly of such fears. Laxatives, cannabis indica, bimeconite of morphia, and a hydrotherapeutic course did him no good. He greatly improved on "Celerina," and at last accounts seemed fairly on the road towards recovery. His digestion and sleep were greatly improved by this preparation, and I consider it aided to make his condition more tolerable ; at the same time I believe he would have recovered, as he had on three previous occasions, without much medication. It is possible, however, that he avoided commitment to an asylum by the use of the medicines prescribed, something terribly humiliating to a proud and sensitive spirit such as that possessed by this gentleman, as well as agonizing to his relatives and friends.

The excessive wear and tear of the muscular system during an attack of chorea and after rapidly recurring epileptic paroxysms, as well as during delirium tremens, would doubtless be diminished by the judicious use of this valuable agent.

I trust the profession may be induced to give this combination of excellent remedies a fair trial in properly selected cases. I know the honorable character of the manufacturers, and that reliance can be placed implicitly upon the purity and genuineness of the agents entering into its composition.

The protest of the profession has always been uttered against "secret nostrums," and "patent preparations," in neither of which category can this combination of well-known, recognized drugs in definite proportions, be classed, nor should the opposition to trade-marks deter any one from its use, when, as in the present instance, the trade-mark is a guarantee of trustworthiness.—*Med. Brief.*

